# Supplementary material for: Hot spot prediction in protein-protein interactions by an ensemble system
Source: BMC Syst Biol. 2018 Dec 31;12(Suppl 9):132. doi: 10.1186/s12918-018-0665-8 (PMC6311905; doi:10.1186/s12918-018-0665-8)
Supplement: Supplementary file 2 — All relevant MATLAB code. A simple Matlab implement of our predictor. (ZIP 19,742 kb) [file 12918_2018_665_MOESM2_ESM.zip › Additional file 2/Read ASA/ASA website/dbMPIKT/1MHPL.html]

 NetSurfP - Protein Surface Accessibility and Secondary Structure Predictions

  

|  |  |  |
| --- | --- | --- |
|  |  | NetSurfP - Protein Surface Accessibility and Secondary Structure Predictions  Technical University of Denmark |

  


---

```
# For publication of results, please cite:
# A generic method for assignment of reliability scores applied to solvent accessibility predictions.
# Bent Petersen, Thomas Nordahl Petersen, Pernille Andersen, Morten Nielsen and Claus Lundegaard
# BMC Structural Biology 2009, 9:51 doi:10.1186/1472-6807-9-51
#
# Column 1: Class assignment - B for buried or E for Exposed - Threshold: 25% exposure, but not based on RSA
# Column 2: Amino acid
# Column 3: Sequence name
# Column 4: Amino acid number
# Column 5: Relative Surface Accessibility - RSA
# Column 6: Absolute Surface Accessibility
# Column 7: Z-fit score for RSA prediction
# Column 8: Probability for Alpha-Helix
# Column 9: Probability for Beta-strand
# Column 10: Probability for Coil
E I  1MHPL                  1    0.355  65.638  -0.757   0.005   0.262   0.733
E Q  1MHPL                  2    0.518  92.568   1.457   0.001   0.959   0.040
B L  1MHPL                  3    0.138  25.231   0.358   0.001   0.959   0.040
E T  1MHPL                  4    0.466  64.690   0.760   0.001   0.900   0.099
B Q  1MHPL                  5    0.207  36.970  -0.182   0.004   0.616   0.381
E S  1MHPL                  6    0.381  44.606   0.151   0.004   0.138   0.858
E P  1MHPL                  7    0.508  72.057  -1.375   0.018   0.019   0.964
E S  1MHPL                  8    0.553  64.858  -1.264   0.018   0.088   0.893
E S  1MHPL                  9    0.365  42.790  -1.314   0.021   0.451   0.528
B L  1MHPL                 10    0.210  38.469  -0.196   0.021   0.756   0.223
E S  1MHPL                 11    0.436  51.146  -0.701   0.018   0.846   0.136
B A  1MHPL                 12    0.178  19.649   0.144   0.021   0.756   0.223
E S  1MHPL                 13    0.395  46.259   0.275   0.004   0.420   0.576
E V  1MHPL                 14    0.505  77.634   0.025   0.004   0.085   0.910
E G  1MHPL                 15    0.684  53.862  -1.379   0.005   0.015   0.979
E D  1MHPL                 16    0.478  68.909   0.321   0.004   0.138   0.858
E R  1MHPL                 17    0.523 119.767   0.871   0.003   0.718   0.279
B V  1MHPL                 18    0.065  10.006   0.050   0.001   0.900   0.099
E T  1MHPL                 19    0.426  59.058   1.596   0.001   0.959   0.040
B I  1MHPL                 20    0.057  10.471   0.115   0.001   0.959   0.040
E T  1MHPL                 21    0.340  47.116   0.872   0.001   0.900   0.099
B C  1MHPL                 22    0.031   4.423  -0.236   0.001   0.900   0.099
E S  1MHPL                 23    0.393  46.036   1.006   0.001   0.900   0.099
B A  1MHPL                 24    0.251  27.693  -1.228   0.002   0.816   0.182
B S  1MHPL                 25    0.317  37.188  -1.137   0.004   0.420   0.576
E S  1MHPL                 26    0.558  65.409  -2.218   0.005   0.045   0.951
E S  1MHPL                 27    0.563  65.960  -2.734   0.005   0.015   0.979
E V  1MHPL                 28    0.392  60.297  -1.518   0.005   0.045   0.951
B N  1MHPL                 29    0.290  42.412  -1.082   0.004   0.197   0.799
B H  1MHPL                 30    0.161  29.322   0.235   0.004   0.616   0.381
B M  1MHPL                 31    0.033   6.663   0.143   0.002   0.816   0.182
B F  1MHPL                 32    0.085  17.160   0.154   0.011   0.918   0.071
B W  1MHPL                 33    0.032   7.792   0.238   0.011   0.918   0.071
B Y  1MHPL                 34    0.049  10.450   0.564   0.011   0.918   0.071
B Q  1MHPL                 35    0.149  26.629   0.667   0.001   0.900   0.099
B Q  1MHPL                 36    0.141  25.218   0.542   0.003   0.718   0.279
E K  1MHPL                 37    0.411  84.440   1.638   0.004   0.138   0.858
E P  1MHPL                 38    0.537  76.186   0.108   0.005   0.015   0.979
E G  1MHPL                 39    0.813  63.975  -2.215   0.003   0.003   0.994
E K  1MHPL                 40    0.503 103.508   0.143   0.018   0.019   0.964
E A  1MHPL                 41    0.390  42.923  -0.265   0.018   0.088   0.893
B P  1MHPL                 42    0.134  19.071   0.139   0.021   0.279   0.699
E K  1MHPL                 43    0.328  67.490   0.704   0.002   0.816   0.182
B P  1MHPL                 44    0.122  17.283   0.521   0.001   0.900   0.099
B W  1MHPL                 45    0.039   9.283   0.438   0.001   0.959   0.040
B I  1MHPL                 46    0.025   4.551   0.168   0.001   0.959   0.040
B Y  1MHPL                 47    0.077  16.412  -0.608   0.001   0.959   0.040
B L  1MHPL                 48    0.085  15.527  -0.548   0.001   0.959   0.040
B T  1MHPL                 49    0.114  15.784  -0.876   0.003   0.718   0.279
B S  1MHPL                 50    0.153  17.990  -1.226   0.005   0.336   0.660
E N  1MHPL                 51    0.476  69.686  -1.379   0.019   0.141   0.840
E L  1MHPL                 52    0.431  78.953  -0.672   0.018   0.088   0.893
E A  1MHPL                 53    0.544  59.916  -1.989   0.018   0.088   0.893
E S  1MHPL                 54    0.666  78.079  -1.874   0.019   0.141   0.840
E G  1MHPL                 55    0.503  39.618  -0.896   0.020   0.205   0.775
E V  1MHPL                 56    0.289  44.481  -0.343   0.019   0.141   0.840
E P  1MHPL                 57    0.511  72.483  -0.950   0.004   0.085   0.910
E S  1MHPL                 58    0.488  57.135  -1.937   0.004   0.138   0.858
E R  1MHPL                 59    0.318  72.936  -0.818   0.004   0.514   0.481
B F  1MHPL                 60    0.082  16.518  -1.231   0.003   0.718   0.279
E S  1MHPL                 61    0.263  30.859  -0.756   0.003   0.718   0.279
B G  1MHPL                 62    0.293  23.075  -2.059   0.021   0.756   0.223
E S  1MHPL                 63    0.378  44.313  -1.395   0.004   0.616   0.381
E G  1MHPL                 64    0.414  32.550  -1.294   0.004   0.420   0.576
E S  1MHPL                 65    0.500  58.635  -1.893   0.004   0.197   0.799
E G  1MHPL                 66    0.577  45.410  -1.983   0.004   0.085   0.910
E T  1MHPL                 67    0.506  70.113  -1.469   0.004   0.197   0.799
E D  1MHPL                 68    0.412  59.355  -0.609   0.004   0.514   0.481
B Y  1MHPL                 69    0.195  41.693   0.035   0.003   0.718   0.279
B T  1MHPL                 70    0.254  35.202  -0.887   0.003   0.718   0.279
B L  1MHPL                 71    0.105  19.299  -0.977   0.003   0.718   0.279
E T  1MHPL                 72    0.272  37.726  -0.322   0.002   0.816   0.182
B I  1MHPL                 73    0.107  19.832  -1.122   0.021   0.756   0.223
E S  1MHPL                 74    0.431  50.478  -0.334   0.022   0.552   0.426
E S  1MHPL                 75    0.498  58.389  -1.260   0.021   0.451   0.528
B L  1MHPL                 76    0.246  44.969  -0.517   0.021   0.279   0.699
E Q  1MHPL                 77    0.477  85.246   0.339   0.020   0.205   0.775
E P  1MHPL                 78    0.329  46.643  -0.654   0.020   0.205   0.775
E E  1MHPL                 79    0.612 106.951  -1.259   0.020   0.205   0.775
E D  1MHPL                 80    0.502  72.281  -0.961   0.004   0.138   0.858
E F  1MHPL                 81    0.366  73.536  -1.174   0.005   0.336   0.660
E A  1MHPL                 82    0.387  42.669  -0.807   0.004   0.514   0.481
E T  1MHPL                 83    0.294  40.764   0.078   0.003   0.718   0.279
B Y  1MHPL                 84    0.058  12.309   0.215   0.002   0.816   0.182
B Y  1MHPL                 85    0.132  28.294   0.702   0.001   0.900   0.099
B C  1MHPL                 86    0.057   7.989  -0.734   0.002   0.816   0.182
B Q  1MHPL                 87    0.145  25.915   0.008   0.023   0.655   0.322
B Q  1MHPL                 88    0.138  24.665  -0.842   0.022   0.552   0.426
B W  1MHPL                 89    0.260  62.626  -1.541   0.022   0.359   0.619
E S  1MHPL                 90    0.407  47.665  -1.521   0.019   0.141   0.840
E G  1MHPL                 91    0.433  34.085  -2.067   0.018   0.088   0.893
E N  1MHPL                 92    0.415  60.727  -1.035   0.018   0.088   0.893
E P  1MHPL                 93    0.388  55.057  -1.424   0.052   0.084   0.864
E W  1MHPL                 94    0.343  82.540  -1.238   0.052   0.084   0.864
E T  1MHPL                 95    0.342  47.408  -1.140   0.056   0.142   0.802
E F  1MHPL                 96    0.364  72.954  -0.961   0.056   0.142   0.802
E G  1MHPL                 97    0.345  27.159  -0.988   0.118   0.150   0.732
E Q  1MHPL                 98    0.332  59.367  -1.075   0.056   0.142   0.802
E G  1MHPL                 99    0.369  29.064  -1.283   0.056   0.142   0.802
E T  1MHPL                100    0.407  56.382  -1.158   0.064   0.216   0.721
E K  1MHPL                101    0.337  69.362  -1.403   0.021   0.279   0.699
E V  1MHPL                102    0.334  51.305  -1.243   0.021   0.279   0.699
E E  1MHPL                103    0.322  56.201  -0.664   0.022   0.359   0.619
E I  1MHPL                104    0.342  63.252  -1.335   0.022   0.359   0.619
E K  1MHPL                105    0.377  77.631  -1.371   0.022   0.359   0.619
E R  1MHPL                106    0.379  86.699  -1.288   0.022   0.359   0.619
E T  1MHPL                107    0.429  59.558  -1.308   0.022   0.359   0.619
E V  1MHPL                108    0.414  63.693  -1.104   0.022   0.359   0.619
E A  1MHPL                109    0.410  45.193  -1.826   0.022   0.359   0.619
E A  1MHPL                110    0.433  47.728  -1.853   0.021   0.451   0.528
E P  1MHPL                111    0.429  60.861  -1.044   0.022   0.359   0.619
E S  1MHPL                112    0.331  38.793  -1.233   0.021   0.279   0.699
B V  1MHPL                113    0.297  45.695  -1.256   0.022   0.359   0.619
E F  1MHPL                114    0.303  60.752  -1.221   0.022   0.359   0.619
E I  1MHPL                115    0.373  69.042  -1.627   0.021   0.451   0.528
E F  1MHPL                116    0.465  93.305  -1.760   0.022   0.359   0.619
E P  1MHPL                117    0.386  54.717  -1.216   0.020   0.205   0.775
E P  1MHPL                118    0.365  51.850  -1.968   0.020   0.205   0.775
E S  1MHPL                119    0.386  45.263  -1.833   0.020   0.205   0.775
E D  1MHPL                120    0.429  61.819  -1.563   0.020   0.205   0.775
E E  1MHPL                121    0.442  77.235  -2.223   0.019   0.141   0.840
E Q  1MHPL                122    0.459  81.906  -1.969   0.018   0.088   0.893
E L  1MHPL                123    0.374  68.406  -1.108   0.018   0.019   0.964
E K  1MHPL                124    0.606 124.736  -0.996   0.018   0.019   0.964
E S  1MHPL                125    0.768  89.963  -0.557   0.003   0.003   0.994
E G  1MHPL                126    0.586  46.110  -1.441   0.005   0.015   0.979
E T  1MHPL                127    0.412  57.158   0.568   0.005   0.336   0.660
B A  1MHPL                128    0.079   8.728  -0.235   0.002   0.816   0.182
B S  1MHPL                129    0.140  16.431   0.540   0.001   0.900   0.099
B V  1MHPL                130    0.033   5.072   0.106   0.001   0.959   0.040
B V  1MHPL                131    0.030   4.580  -0.013   0.001   0.959   0.040
B C  1MHPL                132    0.013   1.797   1.569   0.001   0.959   0.040
B L  1MHPL                133    0.076  13.971   0.311   0.001   0.959   0.040
B L  1MHPL                134    0.025   4.541   0.221   0.001   0.900   0.099
B N  1MHPL                135    0.170  24.947   0.109   0.002   0.816   0.182
B N  1MHPL                136    0.261  38.137  -0.751   0.004   0.514   0.481
B F  1MHPL                137    0.314  63.000  -1.492   0.005   0.336   0.660
B Y  1MHPL                138    0.258  55.113  -0.530   0.004   0.197   0.799
E P  1MHPL                139    0.383  54.291  -1.690   0.004   0.138   0.858
E R  1MHPL                140    0.621 142.095  -2.366   0.004   0.085   0.910
E E  1MHPL                141    0.393  68.587  -1.064   0.005   0.336   0.660
B A  1MHPL                142    0.083   9.114  -0.269   0.003   0.718   0.279
B K  1MHPL                143    0.256  52.762   0.246   0.001   0.900   0.099
B V  1MHPL                144    0.038   5.856   0.052   0.001   0.959   0.040
B Q  1MHPL                145    0.196  34.934   0.981   0.006   0.962   0.032
B W  1MHPL                146    0.039   9.379  -0.400   0.011   0.918   0.071
E K  1MHPL                147    0.252  51.919   0.793   0.001   0.900   0.099
E V  1MHPL                148    0.354  54.333   0.342   0.002   0.816   0.182
E D  1MHPL                149    0.599  86.359   0.059   0.004   0.138   0.858
E N  1MHPL                150    0.656  96.024  -1.367   0.005   0.015   0.979
E A  1MHPL                151    0.704  77.559  -0.596   0.004   0.197   0.799
E L  1MHPL                152    0.621 113.705  -1.681   0.005   0.336   0.660
E Q  1MHPL                153    0.465  83.067  -0.503   0.005   0.262   0.733
E S  1MHPL                154    0.524  61.389  -1.606   0.005   0.262   0.733
E G  1MHPL                155    0.468  36.832  -2.067   0.005   0.262   0.733
E N  1MHPL                156    0.471  68.984  -1.685   0.004   0.420   0.576
E S  1MHPL                157    0.366  42.907  -0.983   0.003   0.718   0.279
E Q  1MHPL                158    0.337  60.117  -1.154   0.002   0.816   0.182
E E  1MHPL                159    0.468  81.829  -1.307   0.023   0.655   0.322
E S  1MHPL                160    0.351  41.149  -1.742   0.021   0.451   0.528
E V  1MHPL                161    0.350  53.780  -0.458   0.021   0.451   0.528
E T  1MHPL                162    0.399  55.383  -1.034   0.022   0.359   0.619
E E  1MHPL                163    0.366  63.958  -0.944   0.021   0.279   0.699
E Q  1MHPL                164    0.519  92.640  -0.634   0.022   0.359   0.619
E D  1MHPL                165    0.545  78.592  -1.928   0.005   0.262   0.733
E S  1MHPL                166    0.601  70.414  -1.675   0.004   0.085   0.910
E K  1MHPL                167    0.655 134.733  -1.592   0.005   0.045   0.951
E D  1MHPL                168    0.720 103.680  -1.050   0.005   0.015   0.979
E S  1MHPL                169    0.354  41.501  -1.491   0.004   0.085   0.910
E T  1MHPL                170    0.319  44.204   0.391   0.004   0.616   0.381
B Y  1MHPL                171    0.091  19.425  -0.338   0.002   0.816   0.182
B S  1MHPL                172    0.099  11.591  -0.716   0.001   0.900   0.099
B L  1MHPL                173    0.067  12.194  -1.021   0.001   0.959   0.040
B S  1MHPL                174    0.111  13.044  -0.357   0.001   0.959   0.040
B S  1MHPL                175    0.040   4.700  -1.038   0.001   0.959   0.040
B T  1MHPL                176    0.179  24.800   0.192   0.001   0.959   0.040
B L  1MHPL                177    0.041   7.562   0.069   0.001   0.900   0.099
E T  1MHPL                178    0.313  43.441   0.684   0.001   0.900   0.099
B L  1MHPL                179    0.062  11.352   0.398   0.003   0.718   0.279
E S  1MHPL                180    0.357  41.829   1.155   0.022   0.359   0.619
E K  1MHPL                181    0.448  92.112  -0.681   0.020   0.205   0.775
E A  1MHPL                182    0.590  65.062  -1.213   0.020   0.205   0.775
E D  1MHPL                183    0.501  72.122   0.521   0.020   0.205   0.775
B Y  1MHPL                184    0.174  37.227  -1.223   0.018   0.088   0.893
E E  1MHPL                185    0.666 116.333   0.307   0.005   0.045   0.951
E K  1MHPL                186    0.642 132.142   0.374   0.005   0.045   0.951
E H  1MHPL                187    0.627 114.015  -0.397   0.005   0.045   0.951
E K  1MHPL                188    0.487 100.114   0.316   0.005   0.262   0.733
E V  1MHPL                189    0.448  68.888   1.766   0.002   0.816   0.182
B Y  1MHPL                190    0.065  13.784   0.867   0.001   0.959   0.040
E A  1MHPL                191    0.321  35.363   1.722   0.001   0.959   0.040
B C  1MHPL                192    0.023   3.285   0.785   0.001   0.959   0.040
E E  1MHPL                193    0.273  47.693   1.382   0.001   0.959   0.040
B V  1MHPL                194    0.073  11.282  -0.355   0.001   0.900   0.099
E T  1MHPL                195    0.430  59.683   0.624   0.001   0.959   0.040
B H  1MHPL                196    0.141  25.630   0.170   0.002   0.816   0.182
E Q  1MHPL                197    0.625 111.643  -0.137   0.005   0.262   0.733
E G  1MHPL                198    0.611  48.078  -1.815   0.005   0.045   0.951
E L  1MHPL                199    0.472  86.387  -0.730   0.004   0.085   0.910
E S  1MHPL                200    0.648  75.992  -0.313   0.004   0.138   0.858
E S  1MHPL                201    0.519  60.839   0.148   0.020   0.205   0.775
E P  1MHPL                202    0.480  68.112   0.233   0.022   0.359   0.619
B V  1MHPL                203    0.225  34.613  -0.182   0.004   0.616   0.381
E T  1MHPL                204    0.572  79.392   0.435   0.003   0.718   0.279
E K  1MHPL                205    0.338  69.424   1.403   0.003   0.718   0.279
E S  1MHPL                206    0.538  63.018   0.516   0.002   0.816   0.182
B F  1MHPL                207    0.087  17.461   0.521   0.002   0.816   0.182
E N  1MHPL                208    0.579  84.692   1.783   0.004   0.616   0.381
E R  1MHPL                209    0.461 105.569   0.620   0.020   0.205   0.775
E G  1MHPL                210    0.636  50.077  -0.914   0.019   0.141   0.840
E E  1MHPL                211    0.656 114.568   1.662   0.018   0.088   0.893
E C  1MHPL                212    0.506  71.028  -1.094   0.003   0.003   0.994

Explain the output.  Go back.
```

---
